# Implementation of a Rapid Response System in a University Hospital: Impact on In-Hospital Mortality and Surgical Patient Outcomes

**DOI:** 10.3390/jcm15093443

**Published:** 2026-04-30

**Authors:** Daiana Toma, Ovidiu Horea Bedreag, Diana Andrei, Marius Păpurică, Claudiu Rafael Bârsac, Adelina Băloi, Alexandru Rogobete, Laura Andreea Ghenciu, Dorel Săndesc

**Affiliations:** 1 Anaesthesia and Intensive Care Research Center, Faculty of Medicine, “Victor Babes” University of Medicine and Pharmacy, 300041 Timisoara, Romania; daiana.toma@umft.ro (D.T.); bedreag.ovidiu@umft.ro (O.H.B.); alexandru.rogobete@umft.ro (A.R.); sandesc.dorel@umft.ro (D.S.); 2 Doctoral School, “Victor Babes” University of Medicine and Pharmacy, 300041 Timisoara, Romania; claudiu.barsac@umft.ro (C.R.B.); adelina.baloi@umft.ro (A.B.); 3 Department of Balneology, Medical Rehabilitation and Rheumatology, “Victor Babes” University of Medicine and Pharmacy, 300041 Timisoara, Romania; 4 Discipline of Pathophysiology, Department of Functional Sciences, “Victor Babes” University of Medicine and Pharmacy, Square Eftimie Murgu 2, 300041 Timisoara, Romania; bolintineanu.laura@umft.ro; 5 Center for Translational Research and Systems Medicine, “Victor Babes” University of Medicine and Pharmacy, Square Eftimie Murgu 2, 300041 Timisoara, Romania

**Keywords:** rapid response team, mobile intensive care team, in-hospital mortality, clinical deterioration, surgical patients, ICU readmission, patient safety, outreach team

## Abstract

**Background/Objectives:** Inpatient clinical deterioration is a major contributor to adverse hospital outcomes, such as unplanned intensive care unit (ICU) admissions and death. Rapid response systems aim to address this challenge by enabling early identification and intervention in at-risk patients. This study evaluated the impact of implementing a mobile intensive care team on clinical outcomes in surgical patients. **Methods:** A retrospective observational cohort study was conducted in a tertiary care hospital, comparing two consecutive periods: a pre-intervention phase (PRETIM) and a post-intervention phase (TIM). The study included 17,156 adult surgical patients. The TIM consisted of a proactive outreach team composed of one attending intensivist and two resident physicians, focusing on post-ICU monitoring and early identification of clinical deterioration on surgical wards. The primary outcome was in-hospital mortality. Secondary outcomes included ICU readmission and length of stay. Multivariable logistic regression adjusted for age, sex and surgical section was performed, along with subgroup and sensitivity analyses excluding early non-modifiable deaths. **Results:** Baseline characteristics were comparable between groups. In-hospital mortality decreased significantly following implementation of the TIM (8.0% vs. 5.3%; *p* < 0.001), corresponding to an absolute risk reduction of 2.7% and a number needed to treat of 37. ICU readmission rates did not differ significantly between groups. Sensitivity analysis excluding early deaths confirmed the mortality reduction. Subgroup analysis demonstrated consistent effects across surgical specialties, with the largest reductions observed in neurosurgery and general surgery. **Conclusions:** The implementation of a mobile intensive care team was associated with a significant and clinically meaningful reduction in in-hospital mortality among surgical patients. The findings support the role of proactive post-ICU monitoring and early intervention strategies in improving patient outcomes in high-risk hospital populations.

## 1. Introduction

Inpatient clinical deterioration represents a substantial concern within healthcare systems, frequently preceding serious adverse events such as cardiac arrest, unplanned intensive care unit (ICU) admissions, or death [1]. Ward-based clinical deterioration is rarely abrupt. Most patients who experience acute clinical worsening or require escalation of care to the ICU show measurable physiological changes in the hours that precede the event [2]. Historically, the problem has not been the absence of warning signs but rather the absence of reliable mechanisms to translate those signs into timely clinical action [3]. Early recognition and intervention can significantly alter patient trajectories, potentially mitigating adverse outcomes. To address this challenge, Rapid Response Teams (RRTs), also referred to as Medical Emergency Teams or Rapid Intervention Teams, were developed to bring critical care expertise to deteriorating ward patients before collapse becomes irreversible [2]. RRTs are multidisciplinary hospital-based teams designed to intervene quickly when hospitalized patients show signs of deterioration [4]. The primary purpose is to prevent failure-to-rescue events, including ICU transfers and cardiac arrests, by identifying and responding to patients at risk before deterioration occurs [5].

The team composition exhibited significant variation across different regions. In the United States, teams were predominantly led by nurses or respiratory therapists. Conversely, in Australia, New Zealand, and Scandinavia, teams were primarily led by physicians. In the United Kingdom, nurse-led teams were the most prevalent [6,7]. In practice, most teams include clinicians with expertise in acute assessment and resuscitation, adapted to local resources [8,9].

Activation rests on a deliberately broad set of criteria: specific thresholds related to heart rate, blood pressure, respiratory rate, oxygen saturation, and level of consciousness are the most commonly codified triggers, but most protocols also permit activation based on a clinician’s subjective concern [10,11,12]. Activation can be mandatory or voluntary. Mandatory activation required calls when predetermined criteria were met, whereas voluntary activation relied on staff discretion [13]. When activated, RRTs performed patient assessment and provided interventions including airway management, respiratory support, cardiovascular support, and medications. Teams were responsible for transfer decisions, including facilitating ICU admission when clinically indicated. The target population comprised ward patients outside the ICU who were experiencing or at risk for clinical deterioration [7,14].

The institutional rationale for creating such teams gained momentum in the late 1990s, following the Institute of Medicine’s landmark report on preventable medical error, which placed avoidable in-hospital deaths at the center of national health policy [15]. The Institute for Healthcare Improvement subsequently named RRT implementation as one of six priority interventions, providing a structured framework that accelerated adoption across hundreds of hospitals [16]. The diffusion of the model beyond North America into European and lower-resource contexts has expanded the evidence base. However, this expansion is characterized by considerable variation in team structure, staffing levels, and the availability of local resources [17,18].

The potential of RRTs has been most rigorously evaluated concerning mortality and cardiopulmonary arrest [15,19]. Additionally, a distinct and arguably more tractable outcome is the prevention of unplanned ICU admissions and readmissions. The effectiveness of RRTs in facilitating early recognition and management of clinical deterioration has been reported across various healthcare settings [20,21].

Despite widespread adoption, the effectiveness of rapid response systems remains variable across studies, partly due to differences in team composition, activation criteria, and institutional implementation. Data on structured, physician-led outreach models focused on post-ICU surgical patients remain limited. The aim of this study was to evaluate the impact of implementing a mobile intensive care team on clinical outcomes in surgical patients in a tertiary care hospital. The study sought to assess whether a proactive outreach model focused on post-ICU monitoring and early identification of clinical deterioration is associated with a reduction in in-hospital mortality, ICU readmission rates, and length of stay.

## 2. Materials and Methods

### 2.1. Study Design and Setting

This study was conducted as a retrospective observational cohort analysis in a high-volume tertiary care hospital. The objective was to evaluate the impact of implementing a mobile intensive care team on clinical outcomes among surgical patients. Two consecutive periods were analyzed: a pre-intervention period (PRETIM), representing standard care, and a post-intervention period (TIM), corresponding to the implementation of the mobile intensive care team. The PRETIM period spanned from January 2023 to December 2023, while the TIM period covered January 2024 to December 2024. The comparison between these two periods allowed assessment of the effect of the intervention under real-world clinical conditions.

### 2.2. Study Population

The study population included all adult patients admitted to surgical departments during the study periods who required hospitalization and had documented clinical outcomes. Patients aged <18 years were not included. Patients without complete outcome data were excluded from the analysis. No additional exclusion criteria based on diagnosis or procedure type were applied. Patients were identified using institutional electronic medical records. No restrictions were applied based on diagnosis. Patients were followed throughout their hospital stay, including transfers between surgical wards and the ICU. Individuals with missing outcome data were excluded from the analysis. Due to the large patient volume and the structure of the available data, detailed clinical variables such as comorbidities, surgical urgency, and perioperative risk scores were not consistently available and could not be included in the analysis.

### 2.3. Intervention: Mobile Intensive Care Team

The intervention consisted of the implementation of a mobile intensive care team composed of one attending intensive care physician and two resident physicians. The team operated with a proactive approach aimed at early identification and management of clinical deterioration. The core function of the TIM was the systematic monitoring of patients following discharge from the ICU, a period known to be associated with increased vulnerability and risk of clinical deterioration. In addition, the team provided support for patients admitted to surgical wards who exhibited signs of instability or were considered at high risk of deterioration, even in the absence of prior ICU admission. Through early clinical assessment and intervention, the team aimed to optimize patient management and prevent progression to critical illness whenever possible. This model represents a shift from traditional reactive care toward a proactive surveillance strategy focused on early intervention.

The TIM operated on a daily basis, typically within the first 24–72 h following transfer to the surgical ward. Additional evaluations were performed upon request by ward staff or when clinical concern was identified. Interventions included clinical assessment, treatment recommendations, escalation of care when necessary, and facilitation of ICU readmission when indicated. The intervention was applied consistently during the TIM period according to institutional practice.

### 2.4. Data Collection and Variable Definitions

Data were extracted from institutional records and included demographic, clinical, and outcome variables. Age was recorded as a continuous variable, and sex was classified as male or female. Length of stay in the surgical ward (LOS section) was defined as the number of days from admission to a surgical department until discharge, transfer, or death. Length of stay in the intensive care unit (LOS ICU) was defined as the total number of days spent in ICU during hospitalization. ICU readmission was defined as any unplanned return to the ICU after initial discharge during the same hospital stay. The primary outcome, in-hospital mortality, was defined as death occurring at any point during hospitalization, regardless of location. Patients discharged alive were classified as survivors. Given the nature of the intervention, particular attention was paid to early deaths. Patients with a surgical ward length of stay of less than or equal to one day were considered to represent cases with immediate or rapidly progressive deterioration, in which the opportunity for intervention was limited. These cases were therefore analyzed separately in a predefined sensitivity analysis.

### 2.5. Outcome Measures

The primary outcome of the study was in-hospital mortality. Secondary outcomes included ICU readmission rates and lengths of stay in both the surgical ward and the ICU.

### 2.6. Statistical Analysis

Descriptive statistics were used to summarize baseline characteristics and outcomes. Continuous variables were reported as means with standard deviations or medians with interquartile ranges, depending on their distribution. Categorical variables were expressed as frequencies and percentages. All percentages are calculated using the total number of patients within each group as the denominator, unless otherwise specified. Comparisons between the PRETIM and TIM periods were performed using appropriate statistical methods. Differences in categorical outcomes, including mortality and readmission rates, were evaluated using risk estimates and corresponding confidence intervals. Missing data were handled using a complete-case approach. Patients with missing primary outcome data were excluded. No imputation techniques were applied due to the retrospective nature of the dataset. The effect of the intervention on mortality was quantified using both relative and absolute measures. Relative risks with 95% confidence intervals were calculated for unadjusted comparisons. Absolute risk reduction was derived as the difference in mortality rates between the two periods, and the number needed to treat was calculated as the inverse of this difference. To assess the independent association between the TIM intervention and mortality, a multivariable logistic regression model was constructed, adjusting for age, sex and surgical section. The results of this analysis were reported as adjusted odds ratios with 95% confidence intervals. The adjusted odds ratios represent the association between the TIM intervention and in-hospital mortality after accounting for potential confounding variables included in the model. Subgroup analyses were performed across surgical specialties to evaluate the consistency of the intervention effect in different clinical settings. Within each subgroup, adjusted logistic regression models were applied. A predefined sensitivity analysis was conducted by excluding patients with a surgical ward length of stay of one day or less, in order to minimize the influence of cases unlikely to be affected by the intervention. A two-sided testing and a significance threshold of *p* < 0.05 was set.

## 3. Results

A total of 17,156 patients were included in the analysis, of whom 8518 were admitted during the PRETIM period and 8638 during the TIM period. The distribution of patients across the included surgical sections was comparable between the two periods, without evidence of major shifts in case volume that could account for outcome differences. Baseline demographic and clinical characteristics are summarized in Table 1. The two groups were well balanced with respect to age and sex. The mean age was almost identical in both cohorts (64.7 ± 8.2 years in the PRETIM group and 64.7 ± 9.1 years in the TIM group, *p* = 0.61), and the proportion of male patients did not differ significantly (54.6% vs. 55.3%, *p* = 0.41). Differences were observed in hospitalization metrics. Patients managed during the TIM period had a significantly longer ward length of stay compared to the PRETIM group (median 7 [4–11] days vs. 4 [2–8] days, *p* < 0.001). In contrast, among patients requiring intensive care, ICU length of stay was significantly shorter during the TIM period (median 2 [1–6] days vs. 3 [1–11] days, *p* = 0.02). The proportion of patients admitted to the ICU was similar between groups (11.8% vs. 12.7%, *p* = 0.07).

**Figure 1:**
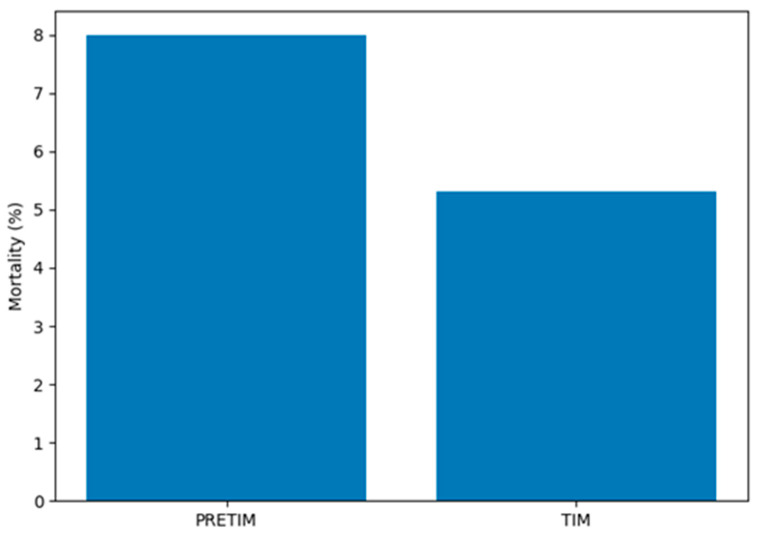
Comparison of in-hospital mortality between the PRETIM and TIM periods. A significant reduction in mortality was observed following implementation of the TIM intervention (8.0% vs. 5.3%; *p* < 0.001).

Clinical outcomes are presented in Table 2. In-hospital mortality was significantly reduced following the implementation of the mobile intensive care team. Mortality decreased from 680 of 8518 patients (8.0%) in the PRETIM period to 458 of 8638 patients (5.3%) in the TIM period (Figure 1), corresponding to a relative risk of 0.66 (95% confidence interval 0.59–0.74, *p* < 0.001). This represents an approximate 34% relative reduction in mortality and an absolute risk reduction of 2.7 percentage points. This corresponds to a number needed to treat (NNT) of approximately 37 patients, indicating that for every 37 patients managed under the TIM strategy, one death may be prevented. To further evaluate the independent effect of the intervention, a multivariable logistic regression model was constructed, adjusting for age and sex. In this model, the TIM period remained associated with a reduction in mortality (adjusted OR 0.80, 95% CI 0.71–0.90). Age remained a significant predictor of mortality, while sex did not demonstrate a significant independent effect. In an expanded multivariable model which included age, sex, and surgical section, TIM implementation was independently associated with lower in-hospital mortality (adjusted OR 0.63, 95% CI 0.55–0.71).

The rate of ICU readmission did not differ significantly between the two periods. Among all hospitalized patients, readmission occurred in 1.2% of patients in the PRETIM group and 1.5% in the TIM group (RR 1.25, 95% CI 0.96–1.62, *p* = 0.09). Similarly, among patients who required ICU admission, readmission rates were 10.1% in the PRETIM group and 11.7% in the TIM group, with no statistically significant difference observed (RR 1.16, 95% CI 0.91–1.48, *p* = 0.27). To account for patients unlikely to benefit from the intervention, a sensitivity analysis was performed excluding individuals who died with a ward length of stay of one day or less, considered to represent early, non-preventable mortality. Following exclusion of these cases, mortality remained significantly lower in the TIM group.

Subgroup analysis by surgical section demonstrated a consistent reduction in in-hospital mortality across all specialties during the TIM period (Table 3). Although the magnitude of effect varied, the direction of association uniformly favored the TIM intervention.

In unadjusted analyses, the most pronounced reductions were observed in higher-risk populations. In neurosurgery, mortality decreased from 16.1% to 9.1%, corresponding to a relative risk of 0.57 (95% CI 0.46–0.71, *p* < 0.001). Similarly, in general surgery III, mortality decreased from 9.4% to 5.7% (RR 0.61, 95% CI 0.47–0.79, *p* < 0.001). Significant reductions were also observed in vascular surgery (7.7% vs. 5.1%, RR 0.66, 95% CI 0.50–0.88, *p* = 0.003) and general surgery II (5.5% vs. 4.2%, RR 0.76, 95% CI 0.57–0.99, *p* = 0.02). Although a reduction was also observed in general surgery I (3.8% vs. 3.0%), this did not reach statistical significance (RR 0.81, 95% CI 0.61–1.07, *p* = 0.23).

After adjustment for age and sex, these findings remained consistent (Table 3). The strongest independent associations were observed in neurosurgery (adjusted OR 0.55, 95% CI 0.44–0.69, *p* < 0.001) and general surgery III (adjusted OR 0.58, 95% CI 0.44–0.76, *p* < 0.001). Significant reductions were also maintained in vascular surgery (adjusted OR 0.64, 95% CI 0.48–0.85, *p* = 0.002) and general surgery II (adjusted OR 0.74, 95% CI 0.56–0.98, *p* = 0.03). In contrast, the reduction observed in general surgery I remained non-significant after adjustment (adjusted OR 0.82, 95% CI 0.61–1.10, *p* = 0.18).

**Figure 2:**
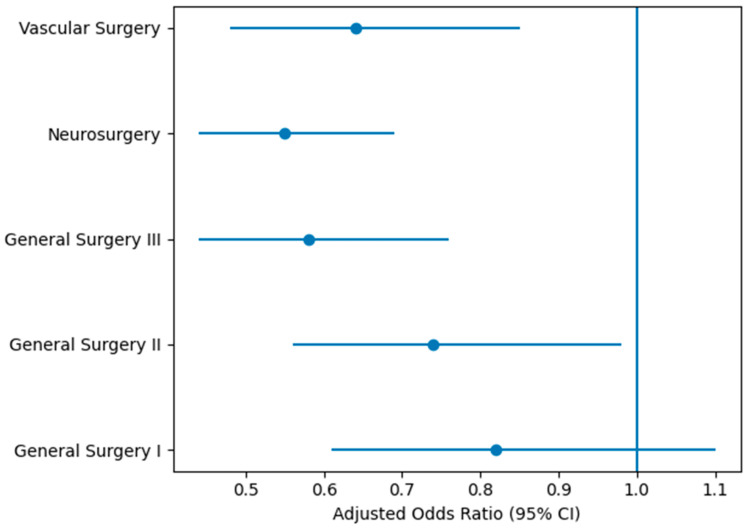
Forest plot of adjusted odds ratios for in-hospital mortality associated with the TIM intervention across surgical sections. Estimates are adjusted for age and sex. The vertical dashed line represents the null effect (OR = 1). Values below 1 indicate reduction in mortality associated with the TIM period.

The consistency of the observed effects across surgical sections is illustrated in Figure 2, which demonstrates the adjusted odds ratios for mortality associated with the TIM intervention across all subgroups.

## 4. Discussion

The present study shows that the implementation of a mobile intensive care team was associated with a significant reduction in in-hospital mortality among surgical patients. This pattern was observed in a large cohort and remained consistent across multiple analytical approaches. The reduction in mortality was not only statistically significant but also of potential clinical relevance, corresponding to an absolute risk reduction of 2.7% and a number needed to treat of approximately 37 patients.

The observed mortality difference may be explained by the proactive nature of the TIM intervention. Unlike traditional reactive models of care, in which deterioration is addressed only after clinical decompensation becomes evident [22,23], the present model facilitates early identification and management of at-risk patients following discharge from the intensive care unit. This approach may reduce delays in recognition of clinical deterioration, optimize treatment escalation, and improve physiological stabilization during a vulnerable transition period. In addition, the involvement of a specialized team may contribute to improved decision-making and closer monitoring, particularly in complex surgical cases.

The reduction in mortality was not accompanied by a significant decrease in ICU readmission rates. This finding may initially appear counterintuitive but can be explained by several factors. First, the intervention may facilitate earlier recognition of deterioration, leading to more rapid and appropriate ICU admissions rather than preventing them altogether. In this context, stable or unchanged readmission rates do not necessarily indicate lack of effectiveness but may instead reflect differences clinical decision-making. Second, the primary benefit of the intervention may lie in preventing progression to irreversible deterioration, rather than reducing the need for ICU-level care per se. An additional finding that warrants consideration is the increase in ward length of stay observed during the TIM period. This may reflect changes in patient management associated with closer monitoring and more cautious discharge practices following ICU stay. Alternatively, it may indicate differences in patient complexity or clinical decision-making between periods. Given the retrospective design, this observation should be interpreted with caution and should not be directly attributed to the intervention.

Studies have shown that the introduction of RRTs were associated with lower rates of in-hospital cardiopulmonary arrest and modest reductions in mortality [7,14]. Meta-analytic evidence similarly indicated a decrease in cardiac arrests and mortality, emphasizing that these benefits are more pronounced when systems are well developed, activation is prompt, and afferent detection is dependable [24]. In another study, ICU readmission rates decreased after implementation of an RRT, suggesting that structured follow-up may improve surveillance for patients recently discharged from ICU [25]. Not all evidence converges on this finding: at some institutions, implementation was associated with increased ICU admission, attributed to a lower threshold for transfer rather than greater illness severity [26]. More recent population-level analysis found that the association between RRT implementation and hospital mortality is not uniformly strong across all settings [27].

The most recent literature suggests that mature, well-resourced models may produce the clearest gains. A retrospective cohort analysis found that a dedicated RRT model was associated not only with lower hospital mortality but also with lower hospital expenditure, indicating that timely rescue may carry both clinical and organizational value [28]. Similarly, work using the Modified Early Warning Score (MEWS) to support RRT decision-making reported improved prediction of ICU readmission risk, illustrating how contemporary rapid response systems increasingly combine bedside expertise with structured surveillance tools [29]. Another study reported that the implementation of a rapid response team was associated with a substantial reduction in inpatient mortality, from 88.93 to 46.44 deaths per 1000 discharges, along with decreases in cardiopulmonary arrest rates [30]. A large multicenter retrospective cohort study conducted across nine hospitals found that rapid response system activation was associated with lower in-hospital mortality, while also highlighting that patients requiring ICU transfer following RRT activation represent a more severely ill subgroup with significantly higher mortality risk [31]. Taken together, the literature supports a balanced conclusion: RRTs are not a single intervention with a uniform effect size, but a complex safety system whose success depends on early detection, credible escalation pathways, dedicated expertise, and continuous institutional learning. However, not all studies have demonstrated consistent improvements in mortality. A large retrospective cohort study found that rapid response team review in the 72 h prior to ICU admission was associated with reduced odds of in-hospital cardiac arrest, but not with a reduction in in-hospital mortality [32].

Despite the demonstrated benefits of rapid response systems, their effectiveness is not uniform across clinical settings, and growing evidence suggests that outcomes are highly dependent on system design, implementation strategy, and institutional context. A scoping review highlighted that team composition, trigger mechanisms, response times, feedback processes, and integration with end-of-life decision-making all vary considerably across institutions, limiting direct comparison but also identifying modifiable design features [3]. Recent integrative reviews have described implementation challenges as structural as well as behavioral: insufficient staffing, unclear protocols, weak data feedback, and limited managerial support can all blunt the potential effect of an RRT even when formally adopted [33,34]. Educational reinforcement is therefore essential. Focused training for RRT members and ward teams has been proposed as a means of improving assessment consistency, communication quality, and confidence during deteriorating-patient events [35].

The findings of this study are consistent with the broader concept of rapid response systems and outreach teams, which have been reported to be associated with to improve patient outcomes through early intervention and enhanced monitoring [36,37]. However, the present study extends this evidence by focusing specifically on post-ICU surgical patients, a population that is particularly vulnerable yet relatively understudied.

The impact of rapid response systems on clinical outcomes remains variable across the literature, with studies reporting inconsistent effects on mortality and ICU-related endpoints [38,39]. This heterogeneity is multifactorial and reflects differences in team composition, institutional organization, resource availability, and the level of engagement of ward staff [12,40,41]. The presence of a dedicated team and the degree of integration within hospital workflows appear to be critical determinants of effectiveness. Variability in activation criteria, response times, and follow-up strategies further contributes to the lack of uniform outcomes reported across studies.

In this context, the structure of the intervention evaluated in the present study may provide important insight into factors associated with improved effectiveness. The mobile intensive care team was composed exclusively of intensive care physicians, allowing for a high level of expertise in the assessment and management of critically ill patients. This differs from many rapid response systems, which are frequently nurse-led or multidisciplinary, with variable team composition [42,43]. The use of a consistent, dedicated team operating within the same clinical framework may have contributed to improved decision-making, reduced variability in clinical assessment, and facilitated rapid and appropriate interventions. These factors may partially explain the magnitude and consistency of the mortality reduction observed in the present study. Such an approach may also contribute to more efficient use of critical care resources. By enabling earlier identification and stabilization of deteriorating patients, a dedicated team may reduce the severity of illness at the time of ICU admission and shorten ICU length of stay, as suggested by the findings of the present study. This is consistent with evidence that well-structured rapid response systems improve both patient outcomes and resource use [3].

Several limitations of this study should be acknowledged. First, the retrospective before–after design of this study introduces a risk of temporal confounding, as the observed differences between the two periods may be influenced by unmeasured confounding factors, temporal trends, or changes in clinical practice over time rather than the intervention alone. Although causal relationships cannot be established, the large cohort provides a quasi-experimental framework for evaluating system-level interventions in routine clinical practice. Second, adjustment in the multivariable analysis was limited to variables available in the dataset. Although the expanded model partially accounts for heterogeneity in case-mix, the absence of more detailed clinical variables remains an important limitation and may result in residual confounding. Diagnostic data were incomplete due to the high volume of patients and the structure of the dataset. As a result, risk stratification was limited, and subgroup analyses were performed primarily at the level of surgical specialty rather than disease-specific categories. This may mask differences in intervention effectiveness across more clinically homogeneous subgroups. However, this limitation reflects real-world clinical practice and does not detract from the observed effect at the population level. Additionally, missing data were handled using a complete-case approach, with exclusion of patients lacking outcome data. Although the proportion of missing data was limited, this approach may introduce selection bias if excluded patients differed systematically from those included in the analysis. Third, the study was conducted in a single center, which may limit external generalizability, although the large and heterogeneous patient cohort mitigates this concern to some extent. Finally, the definition of early non-preventable mortality, based on a surgical ward length of stay of one day or less, represents a pragmatic approach to account for cases with limited opportunity for intervention. While this strategy was supported by sensitivity analyses, it may introduce misclassification bias and may not fully capture all patients with non-modifiable clinical trajectories.

## 5. Conclusions

The implementation of a mobile intensive care team was associated with lower in-hospital mortality among surgical patients, with similar findings observed across multiple analyses. These results suggest that proactive post-ICU monitoring strategies may be associated with improved outcomes in surgical populations, although further studies are needed to confirm these observations.

## Figures and Tables

**Table 1:** Baseline characteristics of surgical patients in the PRETIM and TIM periods.

Variable	PRETIM (n = 8518)	TIM (n = 8638)	*p*-Value
Age, years (mean ± SD)	64.7 ± 8.2	64.7 ± 9.1	0.61
Male sex, n (%)	4652 (54.6%)	4774 (55.3%)	0.41
Ward LOS, days (median [IQR])	4 [2–8]	7 [4–11]	<0.001
ICU admission, n (%)	1004 (11.8%)	1098 (12.7%)	0.07
ICU LOS (ICU patients), days (median [IQR])	3 [1–11]	2 [1–6]	0.02

LOS: length of stay; IQR: interquartile range; ICU: intensive care unit; SD: standard deviation.

**Table 2:** Clinical Outcomes in the PRETIM and TIM Periods.

Outcome	PRETIM (n = 8518)	TIM (n = 8638)	RR (95% CI)	*p*-Value
In-hospital mortality, n (%)	680 (8.0%)	458 (5.3%)	0.66 (0.59–0.74)	<0.001
ICU readmission (all patients), n (%)	101 (1.2%)	128 (1.5%)	1.25 (0.96–1.62)	0.09
ICU readmission (ICU patients), n (%)	101 (10.1%)	128 (11.7%)	1.16 (0.91–1.48)	0.27
Mortality excl. LOS ≤ 1 day (sensitivity), n (%)	↓	↓	Significant	<0.05

RR: relative risk; CI: confidence interval; ICU: intensive care unit; LOS: length of stay.

**Table 3:** Subgroup analysis of in-hospital mortality by surgical section.

Surgical Section	PRETIM Mortality	TIM Mortality	Adjusted OR (95% CI)	*p*-Value
General Surgery I	3.80%	3.00%	0.82 (0.61–1.10)	0.18
General Surgery II	5.50%	4.20%	0.74 (0.56–0.98)	0.03
General Surgery III	9.40%	5.70%	0.58 (0.44–0.76)	<0.001
Neurosurgery	16.10%	9.10%	0.55 (0.44–0.69)	<0.001
Vascular Surgery	7.70%	5.10%	0.64 (0.48–0.85)	0.002

OR: odds ratio; CI: confidence interval. All models adjusted for age and sex.

## Data Availability

The data presented in this study are available on request from the corresponding author. The data are not publicly available due to privacy and ethical restrictions.

## Abbreviations

The following abbreviations are used in this manuscript:

RRT	Rapid Response Team
TIM	Mobile Intensive Care Team
ICU	Intensive Care Unit
LOS	Length of Stay
OR	Odds Ratio
RR	Relative Risk
NNT	Number Needed to Treat
MEWS	Modified Early Warning Score
